# Trained immunity modulators: A new frontier in immunoregulation and disease intervention

**DOI:** 10.1016/j.jare.2025.09.029

**Published:** 2025-09-18

**Authors:** Jiao Chen, Chenxi Wang, Shilin Chen, Hui Cai, Mengke Wang, Jingjie Chang, Xueting Cai, Jie Yang, Peng Cao

**Affiliations:** a Jiangsu Provincial Integrated Innovation Center of Hospital Preparations, Jiangsu Province Academy of Traditional Chinese Medicine, Nanjing, Jiangsu 210028, China; b Jiangsu Provincial Medicinal Innovation Center, Affiliated Hospital of Integrated Traditional Chinese and Western Medicine, Nanjing University of Chinese Medicine, Nanjing, Jiangsu 210028, China; c State Key Laboratory on Technologies for Chinese Medicine Pharmaceutical Process Control and Intelligent Manufacture, Nanjing University of Chinese Medicine, Nanjing, Jiangsu 210023, China; d Shandong Academy of Chinese Medicine, Jinan, Shandong 250014, China

**Keywords:** Trained immunity, Inducer, Suppressor, Vaccine, Polysaccharide

## Abstract

**Background:**

Immunotherapy has emerged as one of the most revolutionary approaches in disease treatments. However, most current and emerging immunotherapeutic strategies primarily target the adaptive immune system. Recent studies have elucidated that innate immune myeloid cells can develop sustained phenotypic modifications upon exposure to specific immunomodulators, mediated through coordinated metabolic alterations and epigenetic reprogramming. These changes lead to either hyperresponsive or hyporesponsive innate immune cells when exposed to secondary stimuli, a phenomenon termed “trained immunity”. Over the past decade, trained immunity has garnered increasing attention for its potential to enhance host defense. While numerous studies have investigated trained immunity modulators, comprehensive reviews—especially those focusing on recently identified modulators—are still lacking.

**Aim of review:**

This review aims to elucidate the molecular mechanisms underlying trained immunity and its dual roles in various pathological conditions. We provide a comprehensive summary of the classifications and mechanisms of trained immunity inducers and suppressors identified in the past decade, emphasizing their therapeutic potential in immune-related diseases. Additionally, we discuss the limitations of these trained immunity modulators and offer insights into future directions for developing novel therapies targeting trained immunity.

**Key scientific concepts of review:** This review first provide an overview of the molecular mechanisms underlying trained immunity, highlighting both its beneficial and detrimental effects on various diseases. Then it focuses on summarizing the classification and mechanisms of trained immunity modulators, including vaccines, polysaccharides, nanobiologics, endogenous mediators and other non-canonical modulators. Finally, the limitations of current trained immunity modulators and insights into future directions for developing novel therapies targeting trained immunity are proposed.

## Highlights

• Trained immunity has garnered increasing attention for its potential to enhance host defense.
• The molecular mechanism of trained immunity and its impacts on various diseases are elucidate.
• The classification and mechanisms of trained immunity modulators, including vaccines, polysaccharides, nanobiologics, endogenous mediators and other non-canonical modulators are summarized in a comprehensive and up-to-date perspective.
• Insights into future directions for developing novel therapies targeting trained immunity are proposed.

## Graphical abstract

**Figure:**
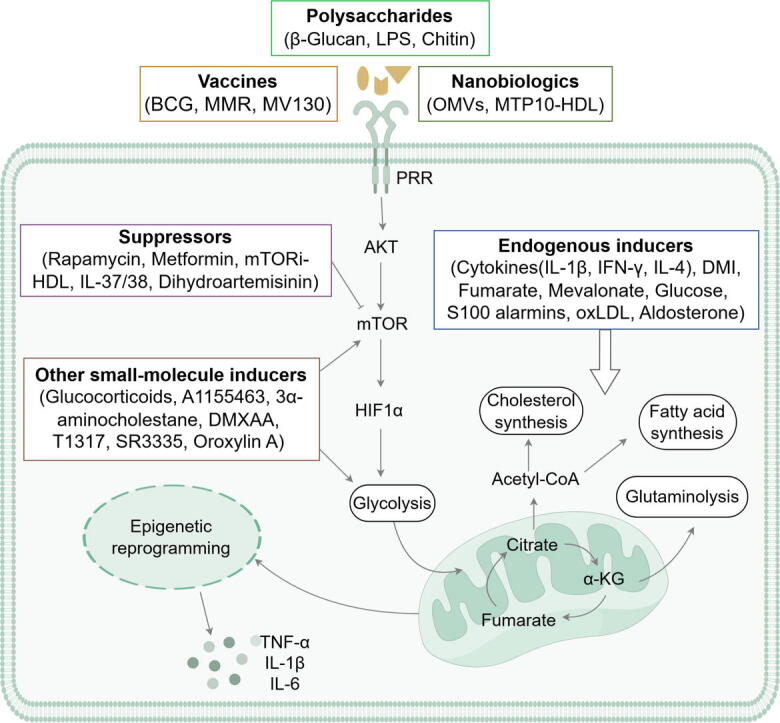

## Introduction

**Fig. 1:**
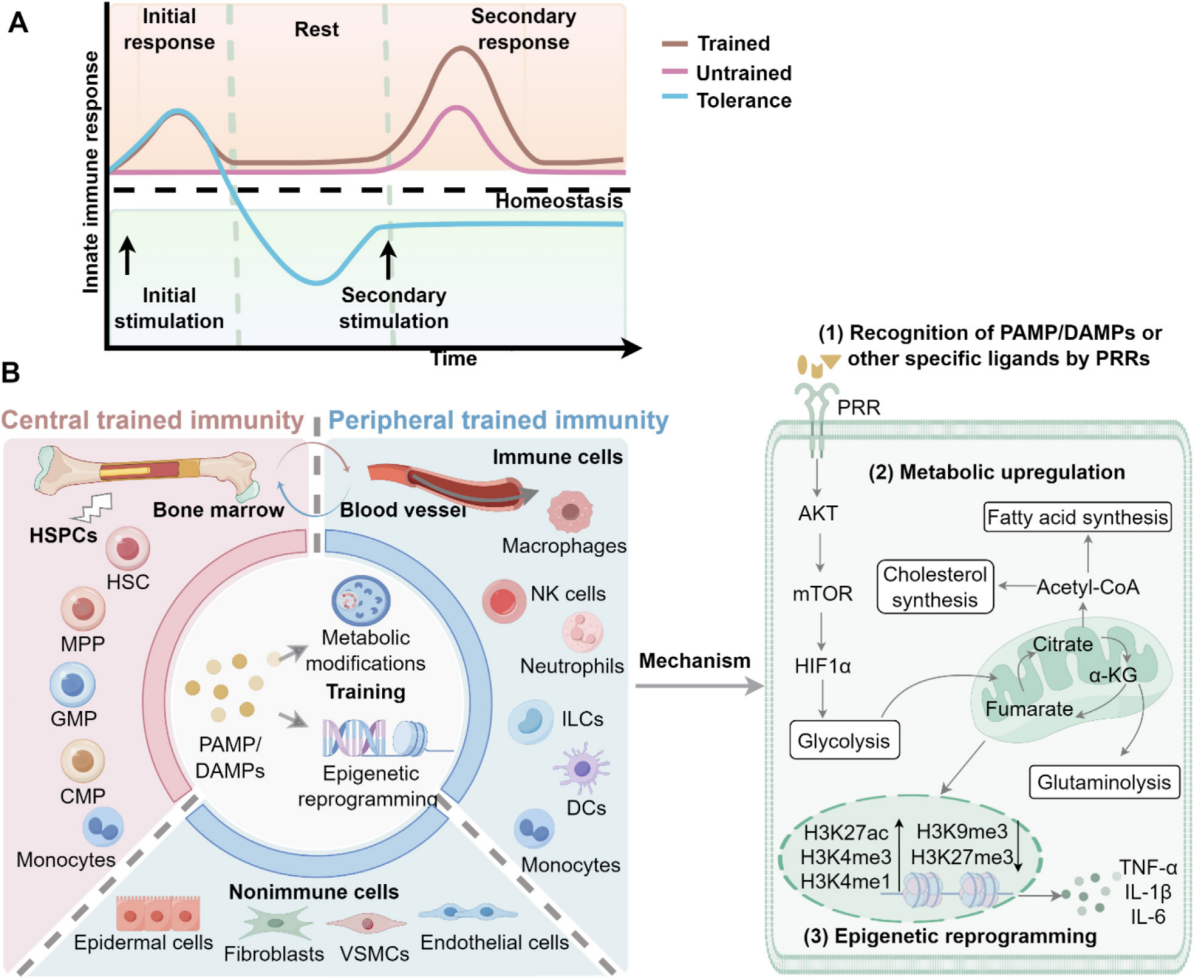
**Trained immunity and involved cell populations.** A. Trained immunity and tolerance. Upon stimulation by immunomodulators, the innate immune cells produce an initial response. Upon secondary stimulation, they produce either hyperresponsiveness (trained immunity) or hyporesponsiveness (tolerance). B. Cell populations and mechanisms involved in trained immunity. PAMP/DAMPs or other specific ligands train the progenitor cells of bone marrow to acquire memory-like phenotypes that are transmitted to circulating cells. Circulating cells, which can also be trained directly by immunomodulators, eventually migrate to tissues, where they enhance proinflammatory responses upon rechallenge. In addition, trained immune reprogramming also occurs in non-immune cells, such as epithelial cells, endothelial cells, VSMCs, fibroblasts, *etc*. Recognition of PAMP/DAMPs by PRRs triggers metabolic upregulation (including glycolysis, TCA cycle, cholesterol/fatty acid synthesis, and glutaminolysis) and epigenetic reprogramming (such as histone demethylation and acetylation), which underlie trained immunity induction. The figure is created with Figdraw.com.

Immune memory has garnered increasing worldwide attention due to its crucial role in the protective efficacy of vaccines and other immunotherapies. For a long time, it was believed that immune memory was a unique feature of adaptive immunity. Consequently, most available immunotherapeutic approaches have primarily focused on the adaptive T lymphocytes, with the most advanced clinical applications including specific antibodies directed against PD-1, PD-L1, and CTLA4, which have brought great benefits to patients [[1], [2], [3]]. However, substantial research in recent decade has challenged this classical view of immune responses, revealing that innate immune cells can also acquire memory-like characteristics after exposure to stimuli. These responses, characterized by either heightened or diminished immune reaction upon secondary exposure to identical or unrelated stimuli, are defined as “trained immunity” (also referred to as “innate immune memory”) and “tolerance”, respectively (Fig. 1 A) [4]. Both phenomena are primarily driven by metabolic and epigenetic reprogramming mechanisms, with divergent outcomes determined by the nature of the priming stimulus and the downstream signaling cascades engaged [[5], [6], [7]].

A prominent example is the Bacille Calmette-Guérin (BCG) vaccine, which is currently approved by FDA for immunotherapy of non-muscle invasive bladder cancer (NMIBC). Notably, its therapeutic effect is largely attributed to the activation of trained immunity [8,9]. Over the past decade, trained immunity has rapidly emerged as a transformative concept in immunology. Given its potential, targeting innate immune cells to regulate trained immunity serves as an encouraging approach for the prevention and treatment of a variety of immune-associated disorders.

Trained immunity represents a durable, functional adaptation of innate immune cells, maintaining regulatory modifications that endure from several months to even years in humans [10,11]. This phenomenon has been observed in both central and peripheral innate immune cells (Fig. 1 B). Early trained immunity research primarily focused on circulating monocytes/macrophages and natural killer (NK) cells [12,13]. Subsequent studies revealed that bone marrow progenitor cells can also acquire memory-like phenotypes and transmit these traits to their offspring, which eventually migrate to tissues and differentiate into tissue macrophages, neutrophils and NK cells [[14], [15], [16]]. More recent findings indicate that trained immunity can also be triggered in other immune cell types such as dendritic cells (DCs) and innate lymphoid cells (ILCs) [17,18]. Interestingly, certain nonimmune cell types, including epithelial cells, endothelial cells, vascular smooth muscle cells, and fibroblasts, have also been shown to acquire trained immunity-like characteristics [[19], [20], [21], [22]].

Unlike T and B lymphocytes, which obtain specific adaptive immune memory though antigen receptor gene rearrangement and clonal expansion, innate immune cells build immune memory mainly via pattern recognition receptors (PRRs). These evolutionarily conserved sensors recognize pathogenic invaders (pathogen-associated molecular patterns, PAMPs) and endogenous danger signals (damage-associated molecular patterns, DAMPs), initiating innate immune activation [[23], [24], [25]]. Major PRRs classes include Toll-like receptors (TLRs), C-type lectin receptors (CLRs), and NOD-like receptors (NLRs), each specialized in detecting distinct microbial or endogenous signals [[26], [27], [28]]. The nature of the PRR-PAMP/DAMP interactions determines the downstream signaling and ultimately the immune responses.

Trained immunity develops mainly through the intricate interactions between metabolic and epigenetic modifications within innate immune cells. Upon initial stimulation, the recognition of PAMPs, DAMPs, or other specific ligands by PRRs triggers cascades of intracellular events that enhance metabolic pathways, including the tricarboxylic acid (TCA) cycle, glycolysis, and cholesterol metabolism, primarily through the AKT-mTOR-HIF1α signaling axis [6,7,[29], [30], [31]]. Metabolites like fumarate and acetyl-CoA, generated through these pathways, regulate epigenetic enzymes, including histone demethylases (*e.g.*, KDM5) and histone acetyltransferases, thereby modifying chromatin structure [6,29]. These processes alter histone methylation and acetylation patterns at promoters and enhancers of proinflammatory genes, increasing chromatin accessibility and facilitating transcriptional activation [32]. As a result, innate immune cells exhibit an enhanced ability to produce cytokines and reactive oxygen species (ROS), as well as increased phagocytic and antimicrobial activity upon re-stimulation [33,34].

From an evolutionary standpoint, trained immunity likely evolves as a mechanism to enhance host defense against recurrent pathogen challenges or tissue damage [4,35,36]. However, maladaptive manifestations of trained immunity, either hyperinflammation or immunosuppression, can contribute to various disease states. Thus, both the induction and deficiency of trained immunity can impact disease pathogenesis and clinical outcomes [37]. In this review, we summarize the beneficial and detrimental effects of trained immunity in different diseases. We also provide an updated overview of potential trained immunity modulators, their mechanisms, and recent advances in trained immunity-based immunotherapy, offering insights for developing novel and more effective modulators for the treatment of related diseases.

## The role of trained immunity in disease

### Protective roles in re-infectious diseases

**Fig. 2:**
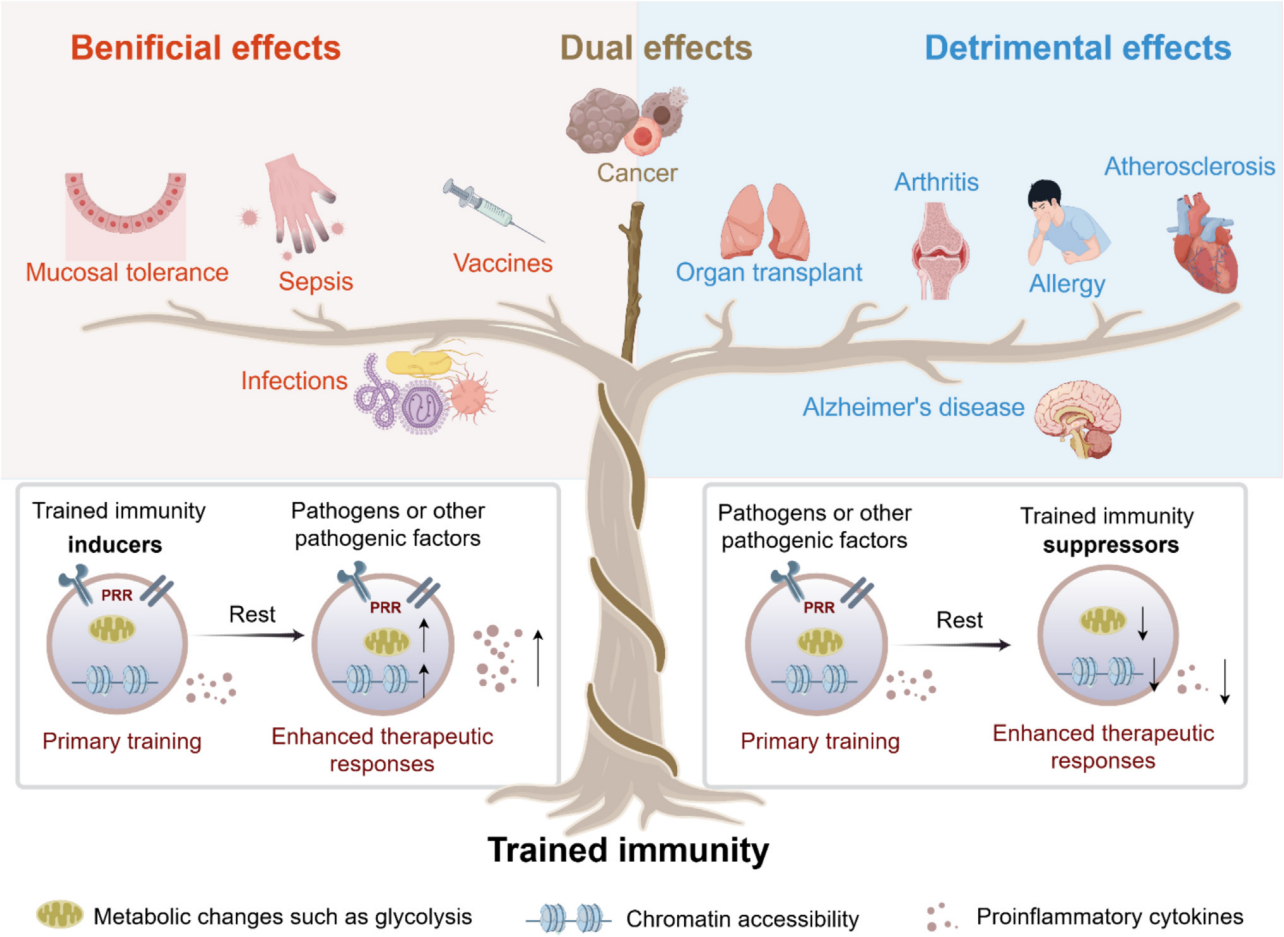
**The dual role and therapeutic prospects of trained immunity**. Trained immunity benefits the treatment of diseases like infection, but also exacerbates the progression of chronic inflammatory diseases like atherosclerosis. Therefore, either inducing or suppressing trained immunity have therapeutic potential. For cancer, properly harnessed trained immunity can induce robust anti-tumor effects, but excessive or chronic stimulation-induced inflammatory states fuel tumor progression. The figure is created with Figdraw.com.

Infectious diseases represent a major public health threat, particularly in low-income regions. Trained immunity, observed in both innate immune cells and certain nonimmune cell types, plays an important role in resisting various exogenous and endogenous pathogens, especially in cases where adaptive immunity is delayed, inefficient or impaired (Fig. 2). This enhanced immune response provides broad and nonspecific protection upon secondary exposure to pathogens, and has been proved particularly beneficial in re-infectious diseases. Early studies found that vaccination with BCG reduced the overall mortality in newborns and low-birth-weight infants [38,39]. Interestingly, BCG vaccination also provides beneficial effects against infections in adolescents and older individuals, highlighting its broad immunostimulatory capacity across different age groups [[40], [41], [42], [43]]. Further studies have revealed that vaccinated with BCG induces cross-immunity, offering protection not only against *Mycobacterium tuberculosis* (Mtb) but also against unrelated pathogens such as influenza virus, poliovirus, *Streptococcus pneumoniae* (*S. pneumoniae*), and *Plasmodium* species [[44], [45], [46], [47], [48]]. This broad-spectrum, T- and B-cell-independent protection is recently attributed to trained immunity. In addition to BCG, other live attenuated vaccines, including the measles-mumps-rubella (MMR) vaccine and oral polio vaccine (OPV), have been shown to induce trained immunity in innate immune system, resulting in broad-spectrum protective responses against subsequent infections, regardless of pathogen specificity [[49], [50], [51]]. This cross-protective effect is particularly beneficial in low-resource settings, where it not only reduces the overall cost of vaccination but also broadens protection against multiple infectious threats.

Beyond vaccines, other trained immunity inducers such as β-glucan, have demonstrated the capacity to enhance protection against a range of heterologous pathogens, including *Pseudomonas aeruginosa* (*P. aeruginosa*), *Staphylococcus aureus* (*S. aureus*), Mtb*,* and *Leishmania braziliensis* (*L. braziliensis*) [7,[52], [53], [54]]. However, persistent or uncontrolled infections can lead to sustained immune activation, resulting in tissue damage. In such cases, excessive trained immune responses may contribute to immunopathology, including sepsis, where overwhelming systemic inflammation leads to multiorgan damage. Therefore, the regulation of trained immunity must be finely tuned to both enhancing host defense and preventing immune-mediated tissue injury.

Throughout the COVID-19 pandemic, the potential of trained immunity-inducing vaccines has been investigated through experimental, clinical and epidemiological studies for their role in protecting against SARS-CoV-2. Animal studies have demonstrated that BCG vaccination provides partial protection against SARS-CoV-2 infection [55,56]. Vaccines such as BCG and MMR are hypothesized to reduce disease severity and mortality associated with COVID-19, although conclusive evidence from large-scale randomized clinical trials is still needed [[57], [58], [59], [60], [61], [62], [63]]. Furthermore, BCG vaccination-induced trained immunity has been demonstrated to heighten the responsiveness of B and T lymphocytes to SARS-CoV-2 vaccines utilizing mRNA and adenoviral vector technologies [57,59]. These findings imply that boosting the innate immune responses may improve vaccine efficacy by creating a more robust immunological environment prior to the activation of antigen-specific responses. Nevertheless, it should be emphasized that, under specific conditions, viral infections themselves can induce overactivation of trained immunity, potentially leading to long-term inflammatory sequelae [[64], [65], [66]]. Lessons learned from trained immunity investigations during the COVID-19 pandemic may provide valuable insights for the design of preventive strategies against future outbreaks of infectious diseases.

### Harmful impact in persistent inflammatory disorders

While trained immunity plays a protective role in combating infections, it also exerts detrimental effects in persistent inflammatory disorders, such as autoimmune disorders, cardiovascular diseases, neurodegenerative conditions, allergies, and organ transplantation rejection (Fig. 2). Under such conditions, maladaptive trained immunity may sustain sterile inflammation and initiate persistent inflammatory cascades, thereby fueling the progression of chronic inflammatory disorders.

In autoimmune disorders such as rheumatoid arthritis (RA), the aberrant production of proinflammatory cytokines can induce trained immunity, leading to heritable modifications of cellular metabolism and gene expression patterns in hematopoietic stem progenitor cells (HSPCs) populations. This reprogramming results in exacerbated inflammatory responses that perpetuate joint inflammation and induce bone destruction [[67], [68], [69]]. Trained immunity has been observed in both mature osteoclasts and their precursors, which produce stronger secondary inflammatory responses and elevated bone resorption in chronic arthritic conditions [70]. As an immunological regulator, trained immunity alters the host's responsiveness to treatment and affects RA outcomes, typically manifesting as persistent inflammatory episodes and relapse following medication withdrawal [68].

The role of trained immunity in atherosclerosis and cardiovascular disease has attracted growing attention. Emerging evidence suggests that trained immunity contributes to the sustained inflammatory response underlying atherosclerotic plaque formation and progression [[71], [72], [73], [74], [75]]. Traditional cardiovascular risk factors such as dyslipidaemia, diabetes, hypertension, along with lifestyle-related factors like stress, insomnia, lack of exercise and Western-style diet, as well as inflammatory states such as infections, gout, and periodontitis, all predispose to induce trained immunity and lead to an elevated risk of atherosclerosis [74]. Moreover, acute adverse cardiovascular events themselves can also cause persistent immunological alterations in HSPCs, contributing to continuous generation of proinflammatory innate immune cells such as monocytes, neutrophils, and NK cells [[71], [72], [73]]. Trained immunity can also occur in tissue-resident innate immune cells, which may infiltrate atherosclerotic plaques, where they contribute to the inflammatory environment and plaque instability.

In the context of neurocognitive disorders, such as Alzheimer's disease (AD), maladaptive trained immunity also exacerbates disease progression. Experimental murine AD models have demonstrated that systemic immune challenges provoke enduring epigenetic reprogramming of central nervous system macrophages (namely microglia), subsequently accelerating cerebral β-amyloid plaque deposition and cognitive decline [76]. These changes are associated with activation of pathways such as HIF1α and mTOR, resulting in transcriptional and functional reprogramming of microglia that promote neuroinflammation [77,78]. Additionally, patients with neurovascular disorders and comorbid systemic inflammation demonstrate trained immunity phenotypes in circulating monocytes, evidenced by hyperresponsiveness to *ex vivo* challenge and elevated release of IL-6/IL-8. This hyperinflammatory profile correlates with both clinical severity and neuropathological progression [79]. These findings indicate that the triggering of trained immunity by chronic systemic inflammation contributes to neurodegeneration via sustained neuroinflammatory responses.

In organ transplantation, post-transplant immune rejection is a crucial factor undermining transplant success, where trained immunity—with its heightened innate immune responses—may potentially compromise graft tolerance. In experimental organ transplantation models, donor organ ischemia–reperfusion injury triggers peripheral inflammation, promoting long-term trained immunity in graft-site macrophages that exacerbates transplant rejection [80]. These trained macrophages exhibit increased pro-inflammatory cytokines production and mTOR/HIF1α pathway activation, which drive changes in epigenetic modification and cellular function. New findings demonstrate that trained immunity impedes kidney transplant success. Uremic toxins such as indoxyl sulfate in patients with chronic kidney disease (CKD) can induce trained immunity, leading to sustained activation of long-lived CD14^+^CD16^++^ memory monocytes [81,82]. These cells promote systemic inflammation and may potentially mediate allograft rejection. Another study establishes trained immunity suppression as a critical determinant of kidney allograft survival, identifying it as a novel therapeutic target to enhance transplant outcomes [83].

### Dual role in cancer progression

Recent research has underscored the great potential of trained immunity in combating cancer. Notably, the BCG vaccine has demonstrated anti-tumor effects through the induction of trained immunity and is currently utilized in the treatment of NMIBC, as well as in certain cases of malignant lymphoma and melanoma [[84], [85], [86]]. In addition, BCG has shown therapeutic potential in hepatocellular carcinoma (HCC) and liver metastases, through mechanisms that are believed to resemble those involved in NMIBC treatment [87]. Moreover, BCG has been associated with a reduced risk of several other malignancies, including lung cancer, leukemia, lymphoma, and colorectal cancers [[88], [89], [90], [91], [92], [93]], although whether the treatment outcomes are directly attributable to trained immunity remains to be fully elucidated.

Another immunostimulatory agent, β-glucan, has also demonstrated the ability to suppress melanoma and Lewis lung carcinoma (LLC) growth by innate immune training of granulopoiesis [33]. Furthermore, whole β-glucan particles have been shown to reprogram lung interstitial macrophages to a trained immunity phenotype, thereby inhibiting tumor metastasis [94]. The strategic enhancement of trained immunity using rationally designed nanobiologics also holds promise in eliciting long-lasting anti-tumor immunity, either as monotherapy or in combination with immune checkpoint blockade therapies (*e.g.*, anti-PD-1) to enhance anti-tumor efficacy [95].

Despite these encouraging findings, trained immunity functions as a double-edged sword in the context of cancer (Fig. 2). On the one hand, properly harnessed trained immunity can induce robust anti-tumor effects; on the other hand, excessive or chronic activation may promote tumor progression through sustained inflammation. Innate immune cells infiltrating the tumor microenvironment (TME) can undergo metabolic and epigenetic reprogramming, resulting in sustained inflammatory responses that may impede cell apoptosis, enhance oxidative stress, promote mitochondrial dysfunction, and ultimately accelerate tumor growth [4]. In particular, cytokines such as IL-6 and TNF-α secreted from trained innate immune cells have been shown to enhance tumorigenesis and metastasis in multiple malignancies [96]. Therefore, a comprehensive understanding of the multifaceted roles of trained immunity in oncogenesis is essential for guiding its therapeutic application.

## Potential modulators of trained Immunity: Inducers and suppressors

**Fig. 3:**
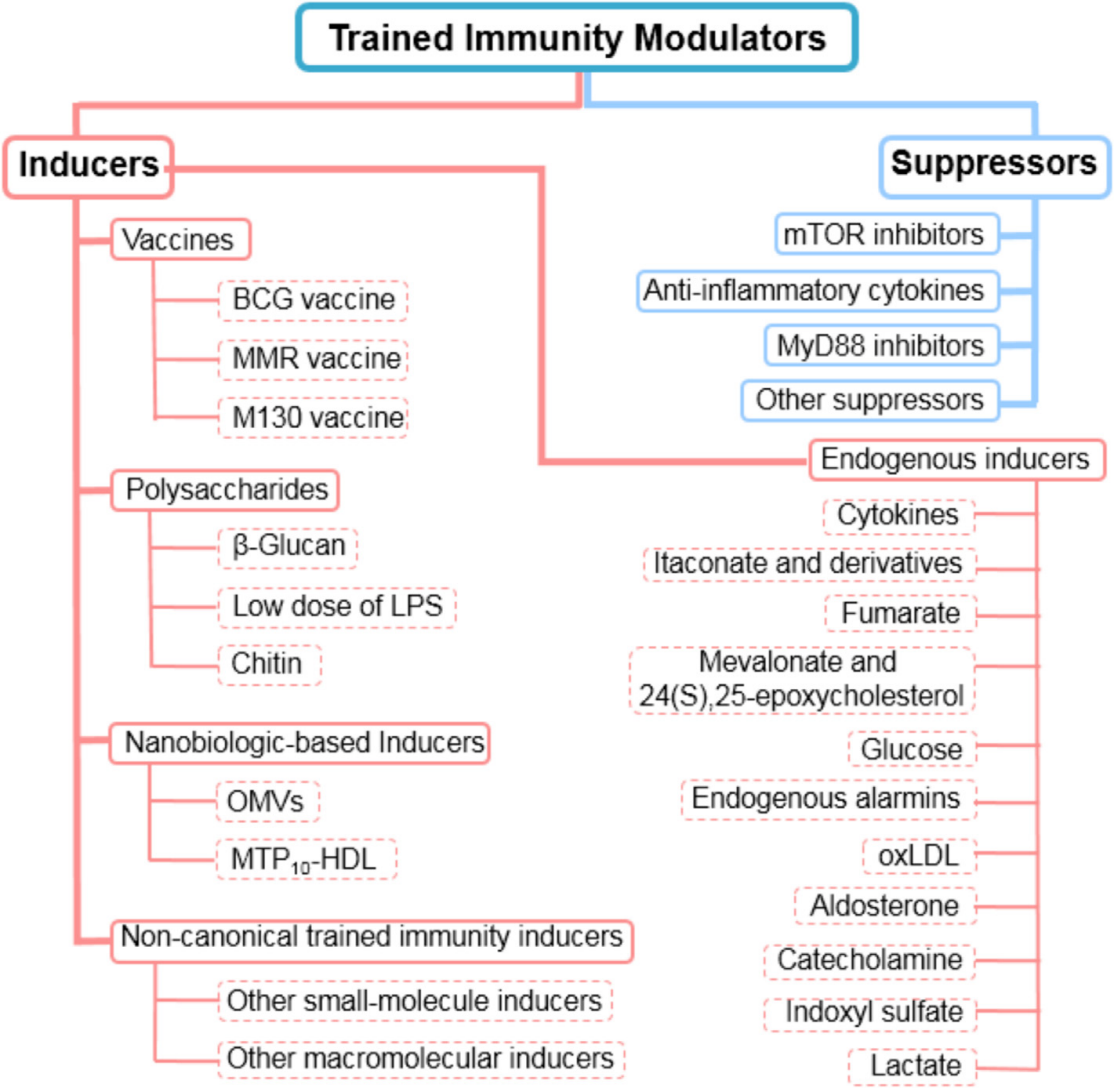
A schematic diagram summarizing the categories and examples of trained immunity modulators.

The factors that regulate trained immunity include immunological modulators (both inducers and suppressors), the gut microbiota and other microbial environments, the inflammatory status, genetic background of the host, and the environmental conditions such as nutrition, lifestyle, and pollutants. This section focuses specifically on the potential inducers and suppressors of trained immunity (Fig. 3), summarizing their types, therapeutic effects, and underlying mechanisms.

### Inducers of trained immunity

The currently identified trained immunity inducers mainly include vaccines, polysaccharides, endogenous metabolites, and certain pathogenic microorganisms. These agents activate trained immunity, producing enhanced and prolonged immune responses upon secondary exposure to either homologous or heterologous stimuli.

#### Vaccines

Unlike traditional vaccines which are designed to stimulate antigen-specific adaptive immune responses, vaccines associated with trained immunity enhance innate immune responses. These vaccines provide “heterologous protection”, extending defense against a wide range of pathogens, irrespective of related or unrelated. Live attenuated viral vaccines such as BCG, MMR, and the polybacterial mucosal vaccine MV130, have demonstrated the ability to induce trained immunity and confer broad protective effects [39,48,[97], [98], [99], [100], [101], [102]]. Additionally, emerging evidence suggests that mRNA vaccines developed against COVID-19 also activate pathways associated with trained immunity [102].

(1) BCG vaccine

Most of our knowledge about trained immunity in human stems from studies on the BCG vaccine, a live attenuated strain of *Mycobacterium bovis* developed for tuberculosis prevention [103,104]. The protective role of BCG against Mtb-unrelated infections and cancers through trained immunity has been discussed in the section of the role of trained immunity in disease. BCG exerts its effects through the activation of PRRs such as TLR2/4 and NOD2, triggering epigenetic and metabolic reprogramming in monocytes and HSPCs. Key molecular features include histone H3K4 trimethylation and enhanced glycolytic activity, contributing to sustained inflammatory cytokine responses [43,105] (Table 1).

**Table 1:** Vaccines, polysaccharides, and nanobiologic-based inducers of trained immunity.

**Types**	**Inducers**	**Reported trained cell types**	**Pathways involved**	**Disease model**	**Clinical status**	**Reference**
**Vaccines**	BCG	Monocytes, macrophages, HSCs	Recognized by TLR2/4 and NOD2, induce autophagy and up-regulate IFN-γ pathway	NMIBC	FDA approved	[40,41,57,58,[106], [107], [108]]
				COVID-19	Phase 4 (Completed)	
	MMR	γδT cells	Boost TNF and IFN-γ production and metabolic pathways	COVID-19	Phase 3 (Completed)	[50,[114], [115], [116]]
	MV130	Myeloid cells	Activate mTOR pathway	Evaluate the ability of inducing trained immunity	Phase 1/2 (Recruiting)	[49,119,120]
**Polysaccharides**	β-Glucan	Macrophages, monocytes, granulocytes, DCs	Interact with Dectin-1 and activate AKT/mTOR/HIF1α pathway, enhance glycolytic and oxidative metabolism	Melanoma	Pre-clinic	[7,33,[52], [53], [54],^94^,[121], [122], [123], [124], [125], [126]]
				Combination therapy for lung carcinoma and neuroblastoma	Phase 1 (Completed)	
				Infectious diseases	Pre-clinic	
	LPS	Macrophages, monocytes, AMs, HSCs	Activate TLR4/MAPK signaling pathway, induce phosphorylation of ATF7 via p38 kinase, increase glycolysis and reduce OXPHOS	Abdominal tumors, combination therapy for melanoma	Phase 1 (Completed)	[[132], [133], [134], [135], [136], [137], [138]]
	Chitin	Monocytes, macrophages, eosinophils	Enhance production of TNF-α and IL-6 through TLR-2 and Dectin-1	Leishmania infection	/	[[143], [144], [145], [146], [147]]
**Nanobiologics**	OMVs	Hematopoietic progenitor cells, macrophages	Activate the inflammasome and TLR-2 signaling pathways	MC38 and B16-F10 tumor models, polymicrobial Sepsis	/	[[148], [149], [150]]
	MTP_10_-HDL	Multipotent progenitors, myeloid cells	Interact with ATP-binding box transporters A1/G1	Melanoma	/	[95]

Beyond infectious diseases, BCG exhibits beneficial effects against cancer, partly medicated by trained immunity [106,107]. Clinical studies report sustained elevation of TNF-α and IL-1β secretion in peripheral monocytes from treated NMIBC patients [106]. Autophagy and interferon (IFNs) signaling are crucial for BCG-induced epigenetic modifications, and their inhibition blocks H3K4 trimethylation in monocytes [9,108].

Single-cell RNA sequencing of immune cells from 156 samples revealed heterogenic transcriptional responses to lipopolysaccharides (LPS) in both monocytes and CD8^+^ T cells. IFN-γ signaling is critical and upregulated in high BCG responders, with *STAT1* identified as a key transcription factor involved in trained immunity in monocytes [108].

To enhance BCG-mediated immune responses, recombinant BCG overexpressing cyclic di-AMP has been developed. This modification improves the efficacy of anti-bladder cancer and exhibits greater potential than wild-type BCG by inducing higher IFN-β production, promoting M1-like macrophage polarization, activating epigenetic markers in proinflammatory cytokine promoters while shifting monocyte metabolism toward glycolysis [109,110].

Combining BCG with cancer immunotherapies has shown promising results [[111], [112], [113]]. A clinical trial found that intravesical BCG combined with MAGE-A3 vaccine therapy enhanced local T-cell responses in patients with NMIBC compared to using the MAGE-A3 cancer vaccine alone [113]. Daman *et al.* proved that intravesical BCG reprogramed HSPCs via IFN-γ, enhancing myeloid cell antigen presentation and promoting T cell infiltration, which synergized with PD-1 blockade [112]. A case report also described disease stabilization in a gastric neuroendocrine carcinoma patient treated with BCG plus nivolumab [111]. However, further large-scale studies are needed to fully evaluate the potential benefits of trained immunity inducers, such as BCG, as adjuvants to immune checkpoint inhibitor therapy.

(2) MMR vaccine

The MMR vaccine provides effective prophylaxis against measles, mumps, and rubella. Epidemiological studies suggest that it also reduces all-cause childhood mortality via heterologous effects [114,115]. A case-control study observed a reduction in COVID-19 incidence among men who received the MMR vaccine [116]. Most research on trained immunity-induced heterologous protection from vaccines such as BCG and influenza has focused on the training of myeloid cells [10,117,118]. However, single-cell multiomics analysis (including transcriptional and epigenetic profiling) from a clinical trial revealed that MMR primarily induced trained immunity in γδT cells rather than monocytes [50]. These γδT cells produce higher levels of TNF and IFN-γ, and upregulate cellular metabolic pathways following MMR stimulation. The differences in trained immunity programs induced by BCG (myeloid cells-dependent) and MMR (lymphoid cells-dependent) suggest that different vaccines can induce distinct types of trained immunity.

(3) MV130 vaccine

MV130 is a whole heat-inactivated polybacterial mucosal vaccine comprising of varying ratios of Gram-positive and Gram-negative microorganisms. Clinical trials report that MV130 significantly reduces the incidence of recurrent respiratory infections [119,120]. In murine models, MV130 preconditioning confers cross-protection against viral respiratory infections [49]. It also protects against systemic candidiasis in a manner independent of B and T lymphocytes, and inhibition of trained immunity through the mTOR pathway abrogates its heterologous protection effect. MV130 induced epigenetic reprogramming of mouse myeloid progenitors, and drives both epigenetic and metabolic alterations in human monocytes, resulting in increased cytokine production [49]. These findings collectively indicate that MV130 is an inducer of trained immunity.

#### Polysaccharides

Extensive evidence highlights the therapeutic efficacy of naturally derived polysaccharides across diverse disease states, demonstrating both prophylactic and interventional benefits. Among them, certain polysaccharides such as β-glucan—an established inducer of trained immunity—have become an extraordinarily popular research topic in the fields of infectious disease and tumor immunology.

(1) β-Glucan

The immunostimulating β-glucan is a natural polysaccharide with (1,3)-linked D-glucose backbone and variable lengths of (1–6) glucosidic side chains. It is abundantly found in the cell walls of plants, fungi and certain bacteria [121]. Recent studies have shown that two forms of β-glucan, soluble fiber β-glucan and whole β-glucan particles (WGP), can induce trained immunity by interacting with Dectin-1 and activating the downstream pathway of Akt/mTOR/HIF1α. The combination of β-glucan with immunotherapy has sparked renewed interest in trained immunity and anti-tumor responses [122].

In the context of cancer, aberrant myelopoiesis often leads to an increased production of myeloid-derived immune cells, such as monocytes, macrophages and neutrophils. These immune subsets typically infiltrate the TME, where they undergo functional reprogramming to acquire distinct pro-tumorigenic characteristics. Kalafati *et al.* demonstrated that prophylactic administration of mice with β-glucan significantly inhibited the growth of melanoma and LLC [33]. The tumor-suppressive properties were mediated by trained immunity mechanisms involving transcriptional and epigenetic remodeling during granulocyte differentiation, as well as the functional polarization of neutrophils from a tumor-promoting phenotype (TAN2) to a tumor-suppressive phenotype (TAN1). These changes were mediated by type I interferon pathway, independent of the host's adaptive immunity.

Pancreatic ductal adenocarcinoma (PDAC) is characterized by insufficient infiltration of activated immune cells in the TME, resulting in limited responsiveness to immunotherapies such as CTLA-4 and PD-1 inhibitors [123]. To overcome the immunologically suppressive microenvironment, particulate WGP β-glucan has been administered intraperitoneally (IP) in murine PDAC models. It selectively accumulates in pancreatic tissue, where it induces infiltration of innate immune cells such as proinflammatory macrophage/monocytes displaying a trained immunity signature in a CCR2-dependent manner [124]. These WGP-educated myeloid cells demonstrate augmented tumoricidal capacity through phagocytic potentiation and ROS-dependent cytotoxicity. In orthotopic PDAC models, WGP-mediated innate immune reprogramming achieves significant tumor regression and survival extension, particularly when combined with PD-L1 checkpoint blockade [124]. Additionally, orally administered β-glucan has been confirmed as an alternative route to elicit trained immunity, working synergistically with surgical ablation to reduce both local and distant PDAC burden [125]. A novel strategy using antibody–β-glucan conjugates (AGC) has been proposed to promote β-glucan delivery into the TME while facilitating interactions between tumor cells and DCs [126]. Compared to monotherapy, AGC induces faster immune responses, increases DC infiltration and T cell activation in the TME, and ultimately leading to enhanced tumor suppression in MC38 murine models.

Tumor metastasis remains the primary cause of cancer-associated deaths, and bone marrow-derived hematopoietic progenitors provide a supportive microenvironment in the pre-metastatic niche [127]. Ding *et al.* discovered that WGP treatment triggers the development of trained immunity characteristics in pulmonary interstitial macrophages, characterized by elevated TNF expression and enhanced phagocytosis upon secondary stimulation [94]. This reprogramming, driven by sphingolipid synthesis and mitochondrial fission, led to reduced metastatic burden and prolonged survival in multiple metastasis models.

In addition to anti-cancer effects, β-glucan also exhibits promising potential in combating infectious diseases. Moorlag *et al.* demonstrated that β-glucan triggers protective trained immunity via histone modifications as evidenced in both human monocytes and murine models of Mtb infection [53]. This protective effect is associated with the expansion of HSPCs and increased myelopoiesis via IL-1 signaling. IL-32 has also been identified to be crucial for β-glucan-induced protection against *L. braziliensis* infection [52]. β-Glucan protects against *S. aureus* sepsis and *Candida albicans* (*C. albicans*) infection via trained immunity mechanisms dependent on mTOR/HIF1α-mediated aerobic glycolysis [7]. Stothers *et al.* found that β-glucan confers protection against *P. aeruginosa* infection in murine models through inducing trained immunity in macrophages, which are characterized by augmented phagocytosis, increased ROS production, and sustained enhanced glycolytic and oxidative metabolism [54]. Superparamagnetic iron oxide (SPIO), a clinical used iron supplement approved by FDA has been shown to relieve LPS-stimulated sepsis in murine models by promoting IL-10 secretion from macrophages. Pan *et al.* developed β-glucan-coupled SPIO nanoparticles (BSNPs), which effectively reprogram macrophages through mTOR signaling to boost trained immunity and enhance bacteria phagocytosis, thereby protecting mice from sepsis and secondary infections [128].

β-Glucan incorporating tumor antigens have been developed as anti-tumor vaccines, aiming to strategically combine trained innate immunity with adaptive immunity for enhanced cancer immunotherapy. In addition to acting as a trained immunity inducer that triggers proinflammatory cytokine production, β-glucan also enhances antigen uptake and presentation by DCs, promotes DC maturation and boosts T cell-mediated immune responses [129]. A recent study developed a personalized cancer vaccine by engineering the model immunogen ovalbumin, β-glucan and the non-viable probiotic *Escherichia coli* (*E. coli*) Nissle 1917 (EcN) [130]. Following subcutaneous injection, the engineered vaccine is phagocytosed by macrophages, resulting in the induction of trained immunity evidenced by enhanced TNF-α production and phagocytosis upon secondary stimulation. These trained macrophages promote DC recruitment and maturation, ultimately enhancing T cell-mediated anti-tumor responses. *In vivo* experiments confirmed that the engineered vaccine efficiently inhibited tumor growth and prevented postoperative tumor recurrence by eliciting stronger adaptive and trained innate anti-tumor immune responses [130].

Another study developed a nano-cancer vaccine incorporating the human papillomavirus (HPV) E7 tumor antigen with two types of trained immunity inducers, β-glucan and muramyl dipeptide (MDP), which almost completely suppressed the growth of TC-1 tumors [131]. The nanovaccine effectively delivers the tumor antigens and trained immunity inducers to the lymph nodes and DCs, promoting DC maturation and augmenting the responses of E7 antigen-specific CD8^+^ T cells expressing IFN-γ [131]. These findings indicate that cancer vaccines adjuvanted with trained immunity inducers hold great promise for improving adaptive immunotherapy, although their efficacy, safety and tolerability in human subjects still require carefully evaluation.

(2) Low dose of LPS

LPS is a structural element of the membranes of Gram-negative bacteria, which functions as a PAMP that binds to TLR4, thereby eliciting trained immunity under specific conditions [132]. Exposure to low concentrations of LPS activates innate immune cells through MAPK/p38 signaling pathway, resulting in elevated secretion of IL-6 and TNF-α upon secondary stimulation [133]. This response is associated with epigenetic changes in myeloid cells, which is durable since LPS stimulation induces persistent H3K4 methylation in corresponding to enhanced responses upon restimulation [134]. LPS-induced trained immunity also depends on the regulation of transcription factors, including ATF7 and C/EBPβ [135,136]. In macrophages, LPS treatment induces phosphorylation of ATF7 via p38 kinase, resulting in ATF7 release from chromatin and the subsequent reduction of inhibitory H3K9me2 histone marks [135]. While in HSCs, epigenetic changes induced by LPS are dependent on C/EBPβ, which facilitates long-term transcriptional changes in gene expression via chromatin remodeling [136]. Moreover, metabolic rewiring accompanies these epigenetic modifications. LPS-activated macrophages exhibit upregulation of glycolysis and downregulation of oxidative phosphorylation (OXPHOS), leading to a significant accumulation of succinate, a metabolite known to promote inflammatory responses [137]. These investigations indicate that LPS can trigger trained immunity through both epigenetic and metabolic pathways. In a related study, Zahalka *et al.* discovered that intranasal exposure to LPS triggers prominent memory responses in alveolar macrophages (AM) upon pneumococcal challenge. This effect is mediated by the type I interferon signaling pathway, fatty acid oxidation (FAO) and glutaminolysis [138].

However, LPS can also induce immune tolerance under different conditions. High concentration of LPS exposure leads to a tolerogenic state characterized by decreased expression of TLR4 and reduced interactions with MyD88, resulting in diminished production of inflammatory cytokines and immunosuppression [133]. Epigenetic modifications are also responsible for LPS-induced immune tolerance, as evidenced by the failure to accumulate activating histone marks at the promoters and enhancers of genes crucial for lipid metabolism and phagocytosis in LPS-treated monocytes [139,140]. Notably, Novakovic *et al*. found that the epigenetic condition of immune tolerance induced by LPS can be reversed by β-glucan treatment [139].

(3) Chitin

Chitin is a natural polysaccharide mainly composed of N-acetylglucosamine units connected by β-1,4-glucosidic bonds [141]. It serves as an important structural component in various organisms, especially in the cell walls of bacteria and fungi, as well as in the exoskeletons of invertebrates such as insects and crustaceans [142]. In mammals, chitin is recognized by the innate immune system, primarily in gut and lung, where it triggers a range of innate immune cells, including monocytes, macrophages and eosinophils, and regulates adaptive immune responses (*e.g.*, Th1, Th2 and Th17 responses). Although the PRRs of FIBCD1, RegIIIc, and NKR-P1 have been reported as chitin-binding receptors, the primary immune recognition of chitin in mammals is mediated through TLR-2/4 and Dectin-1 [143,144]. Interactions with these receptors activate immune cells, leading to cytokine production and the establishment of an immune network that drives inflammatory responses. The full-length chitin polymer is inert; however, catalysis by acidic mammalian chitinase (AMCase) produces bioactive fragment that are recognized by TLR-2 and Dectin-1 on the membrane of immune cells such as macrophages [145]. Rizzetto *et al.* found that fungal chitin triggers trained immunity in monocytes, as evidenced by amplified secretion of TNF-α and IL-6 upon restimulation with TLR ligands in a *Saccharomyces Cerevisiae* infection model [146]. These chitin-trained monocytes also exhibit enhanced intracellular killing ability against fungi and bacteria, with histone methylation playing a key role in this process. Despite promising immunostimulatory properties, the mechanisms underlying chitin-induced trained immunity remain less well understood than those triggered by BCG or β-glucan. In addition to the beneficial effects, chitin-induced trained immunity may also have unexpected detrimental effects, as fungi chitin has been shown to exacerbate the severity of intestinal inflammation in colitis models [147].

#### Nanobiologic-based inducers

In this review, nanobiologics are defined as nanoscale platforms that integrate biological or synthetic carriers (*e.g.*, vesicles, lipoproteins) with trained immunity inducers. Their immunological effects rely not only on the cargo but also on the nanostructure itself, which determines biodistribution, cell targeting, and functional synergy. Over the past decade, researchers have identified highly effective nanoparticles, such as bacterial outer membrane vesicles (OMVs) and MTP_10_-HDL, which target innate immune cells. These nanoparticles can be engineered into nanobiologics by loading them with therapeutic agents.

(1) OMVs

OMVs are nanoscale vesicles naturally secreted by bacteria and contain abundant PAMPs such as peptidoglycan, LPS, and flagellin [148]. These PAMPs endow OMVs with the capacity to function as trained immunity inducers. Pre-inoculation of OMVs to mice activated the inflammasome signaling pathways and stimulated the production of IL-1β. Elevated IL-1β in bone marrow microenvironment causes epigenetic remodeling of hematopoietic progenitor cells, thereby enhancing the production of antigen-presenting cell progenitors for subsequent tumor vaccinations. This process leads to augmented immune responses and enhances the activation of tumor antigen-specific T cells [148]. However, OMVs encounter challenges such as immune tolerance, long-term trained effects, systemic delivery issues, and non-specific tumor-targeting phagocytic activity mediated by macrophages.

To overcome these limitations, OMVs have been engineered into a nanohybrid vaccine by conjugating them with fusion proteins comprising signal regulatory protein-α (SIRPα)-Fc and granulocyte macrophage colony-stimulating factor (GM-CSF) [149]. The OMV-based nanohybrids exhibit enhanced anti-tumor activity in the MC38 tumor model (characterized by tumor-associated macrophage (TAM)-hot and T cell-cold features) through the activation of trained immunity and adaptive T cell-mediated antitumor immunity. In contrast, in the B16-F10 murine melanoma model (characterized by TAM-cold and T cell-hot features), their anti-tumor effects are primarily mediated through trained immunity. TAM-mediated phagocytosis serves as a crucial immunological bridge between innate and adaptive immunity [149]. These findings offer insights into the design of immunotherapies that synergize innate training with adaptive response induction.

Traditional OMVs naturally contain endotoxins such as LPS, which can stimulate both immune responses and reactogenicity. Detoxification strategies have been explored and Gong *et al.* developed BCG–derived OMVs (B-OMVs) [150]. Unlike traditional OMVs, B-OMVs originate from a Gram-positive bacterium, lack LPS, and are non-replicative, thus reducing the risks of LPS-induced tolerance and infection. Their nanoscale properties promote tissue distribution and penetration. B-OMVs demonstrate stronger trained immunity than BCG, driving immune reprogramming and providing robust protection against sepsis in a TLR2-dependent manner. These features highlight B-OMVs as promising candidates for sepsis vaccination and anti-infective therapy.

(2) MTP_10_-HDL

Apolipoprotein A-I (apoA-I), a primary structural protein constituting natural high-density lipoprotein (HDL), interacts with adenosine triphosphate (ATP)-binding box transporters A1/G1 in myeloid cells and has been demonstrated as a carrier to form nanobiologics targeting trained immunity. Priem *et al.* developed MTP_10_-HDL nanobiologics by encapsulating MTP (a TLR agonist) within apoA-I-derived HDL-like particles. These nanobiologics showed promising anti-tumor effects in a mouse melanoma model [95]. MTP_10_-HDL exhibits high avidity for bone marrow and can induce myelopoiesis via trained immunity, driven by epigenetic reprogramming of multipotent progenitors. This process helps overcome the immunosuppressive TME, thereby enhancing immune surveillance and effector function. Furthermore, MTP_10_-HDL-promoted trained immunity enhances the therapeutic effectiveness of immune checkpoint blockade therapies targeting PD-1 and CTLA-4, suggesting a potential synergistic strategy for cancer immunotherapy. ApoA-I also holds potential for loading with trained immunity inhibitors, such as mTOR inhibitor rapamycin and S6 kinase-1 (S6K1), which may augment the innate immunosuppressive effects.

#### Endogenous inducers

(1) Cytokines

Trained immunity is characterized by enhanced proinflammatory responses when the host encounters secondary stimuli, making proinflammatory cytokines a crucial component in this process. The IL-1 cytokine family has been established as a critical mediator of trained immunity, especially in the context of β-glucan stimulation and BCG vaccination. Inhibition of IL-1 counteracts the enhanced glycolysis and myelopoiesis induced by β-glucan. Furthermore, the protection efficacy of β-glucan against Mtb infection is abolished in IL-1 receptor-deficient murine models [15,53]. Arts *et al.* observed a strong correlation between elevated production of IL-1β and reduced viremia in individuals immunized with BCG followed by yellow fever vaccination as a secondary immunological challenge, indicating a functional role for IL-1β in enhancing antiviral defense [151]. Genetic variations in the *IL1B* locus, particularly single nucleotide polymorphisms (SNPs), have been shown to influence the magnitude of BCG-induced trained immune responses. Another study from Simats *et al.* demonstrated that IL-1β drives post-stroke trained immunity via epigenetic reprogramming of myeloid cells, and therapeutic inhibition of either IL-1 signaling or monocyte recruitment preserves cardiac function after ischemic injury [72]*.* Importantly, IL-1β is validated to induce trained immunity in both human hematopoietic progenitor cells and monocytes *in vitro* through immunological and epigenetic mechanisms [151,152]. The mechanism underlying IL-1β-induced trained immunity may depend on HIF1α, a transcription factor activated via the AKT/mTOR signaling pathway, which plays a pivotal role in trained immunity establishment [7,137].

In addition to the IL-1 family, IFNs, particularly IFN-γ, have emerged as critical regulators of trained immunity [9,16,33,108,153,154]. BCG-trained HSCs give rise to epigenetically altered monocytes/macrophages that exhibit heightened protection against Mtb infection. These protective effects are contingent upon type II IFN (IFN-γ) signaling [16]. Conversely, Mtb can subvert trained immunity by reprogramming HSCs through type I IFN (IFN-α and IFN-β)/iron axis, which inhibits myeloid cell differentiation and impairs the protective trained immunity [153]. Further insights from Tran *et al.* indicates that BCG-induced CD4^+^CX3CR1^hi^ T cells are essential for cross-protection and serve as potent producers of IFN-γ [154]. Remarkably, IFN-γ alone is sufficient to educate AMs to generate long-term memory, thereby enhancing protection against influenza A viruses (IAV) infection in mice.

Interestingly, IL-4, a well-known anti-inflammatory cytokine, has been shown to exhibit dual roles. Schrijver *et al.* revealed that while IL-4 suppresses acute inflammation via STAT6-dependent signaling, it simultaneously induces trained immunity in monocytes through activation of the PI3K–mTOR pathway and enhanced OXPHOS, indicating a previously underappreciated proinflammatory function [155,156]. Given the unfavorable pharmacokinetic characteristics of recombinant IL-4, an engineered fusion protein comprising apoA-I and IL-4 embedded in nanoparticles has been developed. This IL-4 nanotherapy counteracts immune tolerance in mice with LPS-induced hyperinfammation, as well as in *ex vivo* models of human sepsis and endotoxemia [156]. Therapeutic agents with dual effects—simultaneously reducing inflammation and overcoming immune tolerance—may facilitate patient recovery and help prevent secondary infections.

Other cytokines, including IL-12/15/18, IL-32, IL-36γ, and GM-CSF [[157], [158], [159], [160], [161], [162], [163], [164], [165], [166]], have also been highlighted as essential in triggering and maintaining trained immunity (Table 2). Their roles in orchestrating immunological memory within the innate compartment highlight the complex regulatory network underpinning this phenomenon.

(2) Itaconate and its derivatives

**Table 2:** Cytokines involved in trained immunity.

**Effect on** **trained immunity**	**Cytokine**	**Secreted by**	**Characteristic**	**Trained cell types**	**Pathways**	**Reference**
**Inducers**	IL-1β	Monocytes, macrophages, DCs	proinflammatory	Monocytes, myeloid cells, macrophage	AKT/mTOR/HIF1α pathway	[7,8,15,53,137,151]
	IFN-γ	CD4^+^ T cells, CD8^+^ T cells, NK cells, CD4^+^CX3CR1^hi^ T cells	proinflammatory	HSCs, monocytes, macrophages	/	[9,33,108,153,154]
	IL-4	Th2 cells, monocytes, mast cells	anti-inflammatory	Monocytes, macrophages	PI3K-mTOR, OXPHOS	[155,156]
	IL-12/15/18	Macrophages, DCs, NK cells	proinflammatory	NK cells	Akt/mTOR/HIF1α pathway	[157,158]
	IL-32	Monocytes, epithelial and endothelial cells	proinflammatory	HSCs	/	[159]
	IL-36γ	Monocytes, T cells	proinflammatory	monocytes	NF-κB and mTOR signaling	[160]
	GM-CSF/IL-3	T cells, monocytes/macrophages	/	HSPC, monocytes	p38 and SIRT2	[161,162]
**Suppressors**	IL-10	Regulatory T cells, B cells, *etc*.	anti-inflammatory	Monocytes, myeloid cells, HAECs	Inhibit STAT3 pathway and ROS production	[163,164]
	IL-35	Regulatory T cells, B cells, *etc*.	anti-inflammatory	HAECs	Inhibit ROS production	[163]
	IL-37	Monocytes, macrophages, DCs	anti-inflammatory	Monocytes, BMDMs	Inhibit mTOR signaling and IL-1β processing	[165]
	IL-38	Monocytes, macrophages, DCs	anti-inflammatory	Monocytes, BMDMs	Inhibit AKT/mTOR/S6K signaling	[158,166]

Itaconate is an immunoregulatory metabolite synthesized by immune cells that elicits anti-inflammatory and antioxidant reactions. It plays a pivotal function in processes including immune regulation, host defense, and tumorigenesis. Under proinflammatory conditions, the enzyme immune-responsive gene 1 (IRG1) is upregulated, catalyzing the decarboxylation of *cis*-aconitate (a TCA cycle intermediate) to itaconate [167]. A study by Domínguez-Andrés *et al.* showed that acute activation of myeloid cells by LPS stimulation resulted in a marked increase in IRG1 expression, leading to extensive accumulation of itaconate. This accumulation blocks succinate dehydrogenase (SDH) activity and contributes to eliciting innate immune tolerance in monocytes [168]. Notably, β-glucan has been shown to suppress IRG1 expression and restore SDH activity in a human endotoxemia model, thereby counteracting LPS-induced tolerance by preserving the integrity of the TCA cycle. This indicates that the itaconate biosynthetic pathway serves as a crucial metabolic checkpoint in the balance between immune tolerance and trained immunity [168].

In contrast, the study by Ferreira *et al.* reveals that itaconate and its derivative dimethyl itaconate (DMI) initially exhibit short-term immunosuppressive effects but ultimately induce long-term trained immunity [169]. After five days of exposure to itaconate or DMI, LPS-stimulated human monocytes showed increased secretion of proinflammatory cytokines. DMI also induces epigenomic and metabolic alterations, regulates IFN responses and enhances ROS production, similar to the effect of β-glucan. The induced trained immunity was found to be dependent on glutathione synthesis. Moreover, *in vivo* investigations demonstrated that DMI treatment protected mice from *S. aureus* infection even in the absence of additional stimuli [169]. Taken together, these findings suggest that while itaconate and its derivatives may exert immunosuppression properties under acute proinflammatory conditions, they can also induce trained immunity in the absence of further stimulation. Nevertheless, the precise immunomodulatory mechanisms remain incompletely understood. Given the potential to trigger complex immune responses, caution is warranted when considering therapeutic applications of itaconate and its derivatives.

(3) Fumarate

Fumarate is a critical metabolic intermediate in TCA cycle generated through the catalysis of succinate by SDH in mitochondria. Arts *et al.* found the accumulation of fumarate in β-glucan-trained monocytes, which establish a link between immunometabolic reprogramming and long-term epigenetic modifications, notably by suppression of histone demethylase KDM5 [6]. Fumarate also prevents the degradation of HIF1α by inhibiting its hydroxylation, which can enhance the responses induced by β-glucan [6,7,137]. Importantly, fumarate itself has been shown to trigger trained immunity by enhancing trimethylation of histone H3 at lysine 4 (H3K4me3) and acetylation at lysine 27 (H3K27ac), recapitulating key aspects of β-glucan-mediated epigenetic reprogramming [6]. Another study also confirmed that extracellular fumarate treatment increased secretion of TNF-α upon LPS stimulation in monocytes [170]. Furthermore, fumarate induces mitochondrial fusion both *in vitro* and *in vivo*, which is critical to the initiation of trained immunity in monocytes. Fumarate supplementation has been demonstrated to enhance mitochondrial fusion in both intestinal cells and muscle tissue, and enhance resistance to pharyngeal infection by *E. coli* in *C. elegans*. Collectively, these findings underscore a significant association between mitochondrial dynamics and fumarate-induced trained immunity.

(4) Mevalonate and 24(S),25-epoxycholesterol

Recent studies indicate that activation of the cholesterol biosynthesis pathway, particularly the production of mevalonate, serves as a critical regulator in the process of trained immunity in myeloid cells. This effect is mediated via the stimulation of the insulin-like growth factor 1 receptor (IGF1-R) and mTOR signaling, which in turn induces histone alterations such as H3K27ac enrichment in inflammatory pathways [30]. Notably, mevalonate accumulation resulting from mevalonate kinase deficiency leads to a persistently trained immunological state in monocytes from hyperimmunoglobulin D syndrome (HIDS) patients, observed at both immunological and epigenetic levels. This phenomenon may explain the recurrent inflammatory episodes observed in HIDS patients. Conversely, statins, pharmacological inhibitors of hydroxymethylglutaryl-CoA (HMG-CoA) reductase that reduce mevalonate biosynthesis, can disrupt immune system homeostasis [171]. Notably, blockade of mevalonate biosynthesis has been associated with proinflammatory consequences, highlighting its dual immunomodulatory role.

Furthermore, the shunt pathway of cholesterol synthesis catalyzed by squalene epoxidase (SQLE) produces 24(S),25-epoxycholesterol (24(S),25-EC), a metabolite essential for β-glucan-triggered trained immune responses in macrophages. 24(S),25-EC stimulates liver X receptor (LXR) and increases chromatin openness, thereby inducing epigenetic reprogramming associated with trained immune memory. Simultaneously, 24(S),25-EC production promotes ROS accumulation and stabilizes HIF1α, facilitating metabolism conversion towards glycolysis. Pretreatment of macrophages with 24(S),25-EC significantly augments the production of TNF-α, IFN-β, and IFN-γ upon secondary stimulation [172]. Moreover, 24(S),25-EC-induced trained immunity has been shown to promote anti-tumor activity *in vivo*, offering promise for future immunotherapeutic applications.

(5) Glucose

Hyperglycemia is a potential driver of atherosclerotic cardiovascular diseases. Even with rigorous glycaemic control, hyperglycemia-related complications persist in diabetic patients, indicating the existence of “hyperglycemic memory” [173]. The phenomenon can be attributed to epigenetic remodeling of monocytes and HSCs [74,173,174]. Experimental studies have demonstrated that elevated glucose concentrations enhance proinflammatory gene expression in monocytes/macrophages through a glycolysis-dependent mechanism. The induced trained immunity subsequently promotes the progression of atherosclerosis in mice [71]. Bone marrow transfer from diabetic mice to normoglycemic *Lldr*-deficient recipients exacerbates atherosclerotic plaque development, confirming the persistence of hyperglycemia-induced trained immunity. The phenotype in HSCs is marked by alterations of H3K4me3 and H3K27ac levels and chromatin accessibility at pro-inflammatory genes. The open chromatin is associated with Runt-related transcription factor 1 (RUNX1), as its inhibition reverses this trained state [71]. These observations elucidate the pathways through which diabetes-associated proinflammatory states contribute to the development of various complications, particularly atherosclerosis.

(6) Endogenous alarmins

S100 alarmins, an important class of DAMPs, play essential roles in immune regulation and inflammatory disease pathogenesis. Preconditioning monocytes with S100A4 enhances their response to LPS re-stimulation, resulting in enhanced secretion of inflammatory mediators including IL-6 and TNFα [175]. This S100A4-induced trained immunity is mediated by a PRDM8-dependent mechanism. Ulash *et al.* have demonstrated that low concentrations of S100 alarmins in neonatal plasma are significantly associated with high susceptibility to neonatal sepsis. S100A8/A9 treatment protects mouse neonates from fatal sepsis, highlighting their immunomodulatory potential [176]. As TLR4 agonists, high level of S100A8/A9 alarmins induce trained immunity in human monocytes by promoting MyD88-dependent proinflammatory genes expression such as *IL6*, *IL1B* and *TNF*. Notably, they also promote a phenomenon known as “stress tolerance”, wherein phagocytes become unresponsive to a secondary TLR4 stimulus, thus mitigating excessive inflammatory damage [177]. This immune adaptation protects newborns by preventing overzealous inflammatory responses, thereby enhancing their resistance to inflammatory and septic diseases.

Another type of endogenous alarmin, cathelicidins, are host-derived antimicrobial peptides constituting part of the innate immune system. van Dijk *et al.* demonstrated that monocytes trained with DCATH-2, a cathelicidin analog, exhibit enhanced proinflammatory responses and antimicrobial killing capacity [178]. The DCATH-2-induced trained immunity is mediated by purinergic receptors and depends on activation of the PI3K-mTOR-HIF1α axis and p38 MAPK signaling pathway.

(7) Oxidized low-density lipoprotein

Oxidized low-density lipoprotein (oxLDL) plays a crucial role in both the initiation and chronic progression of atherosclerosis, in part by inducing a proinflammatory state of trained immunity in monocytes and macrophages [19,179,180]. After exposure to oxLDL, human monocytes undergo metabolic reprogramming through activation of the mitochondrial metabolic pathway, leading to a heightened state of responsiveness. This transformation is reflected not only by the enlargement of mitochondrial morphology and increased OXPHOS activity, but also by the accumulation of TCA cycle metabolites and the engagement of alternative pathways such as those involving glutamine and free fatty acids metabolism [179]. Such reprogramming provides a sustained energy source for oxLDL-trained macrophages, thereby enhancing their cytokine production capacity and inflammatory responses. Moreover, LXR, a key regulator of both metabolism and inflammation, is critically involved in oxLDL-induced trained immunity. Activation of LXRα specifically amplifies the inflammatory responses induced by oxLDL, while its inhibition blocks both epigenetic reprogramming and metabolic changes of trained immunity [180]. Furthermore, mTOR-dependent ROS production has been identified as a driver of this phenotype, highlighting potential therapeutic targets for atherosclerosis [19].

(8) Aldosterone

Aldosterone, a steroid hormone secreted by the adrenal cortex, is associated with increased cardiovascular risk when dysregulated. It promotes the generation of ROS in macrophages through the mineralocorticoid receptor (MR) upon restimulation [181]. Interestingly, aldosterone-trained macrophages exhibit no significant increase in glycolysis or OXPHOS activity, distinguishing from β-glucan- or BCG-trained immunity [25]. Additional research revealed that aldosterone upregulates the epigenetic marker H3K4me3 at the promoter sites of key fatty acid biosynthesis genes such as *FASN*, *ACACA* and *ELOVL6* [25]. This observation implies that the trained immunity established through aldosterone *in vitro* depends on epigenetic regulation of lipid metabolism. The resultant pro-inflammatory macrophage phenotype contributes to cardiovascular complications, implicating aldosterone-induced trained immunity in chronic disease development.

(9) Catecholamine

Catecholamines, including adrenaline and noradrenaline, are pivotal neurotransmitters in the nervous system, which are primarily released under physical and emotional stress. High levels of catecholamines have been implicated to contribute to inflammatory monocytosis and atherosclerosis via interactions with adrenergic receptors [182]. Further study reveals that exposure of human monocytes to adrenaline and noradrenaline results in acute immunosuppressive effects followed by long-term trained immunity, as demonstrated by enhanced secretion of TNF-α and IL-6 upon LPS restimulation [12]. This catecholamines-induced trained immunity is mediated by the β-adrenergic receptor-cAMP pathway, alongside metabolic reprogramming involving augmented glycolysis and OXPHOS. In pheochromocytoma/paraganglioma (PHEO) patients characterized by persistently high catecholamine levels, monocytes exhibit transcriptional upregulation of inflammatory genes and H3K4me3 enrichment at key loci. This *in vivo* evidence confirms catecholamine-induced trained immunity and may explain the high cardiovascular disease burden observed in PHEO patients [12].

(10) Indoxyl sulfate

Indoxyl sulfate (IS), a uremic toxin found at elevated concentrations in patients with chronic kidney disease (CKD), contributes to systemic inflammation and cardiovascular complications. Kim *et al.* demonstrated that IS elicits trained immune responses in human monocytes via the aryl hydrocarbon receptor (AhR)-mediated arachidonic acid (AA) signaling cascade [82]. Moreover, IS-induced trained immunity has been confirmed in both *ex vivo* and *in vivo* models, implying its critical role in mediating chronic inflammatory responses in CKD patients. These findings position IS as a potential therapeutic target for managing inflammation and complications associated with CKD.

(11) Lactate

Lactate, traditionally viewed as a metabolic byproduct, is now recognized as a crucial signaling molecule in activating trained immunity. Recent studies reveal that lactate supports metabolic reprogramming of monocytes and macrophages by fueling the TCA cycle, thereby enhancing their long-term responsiveness to subsequent challenges [183,184]. Trained monocytes are confirmed to preferentially utilize lactate over glucose as a TCA cycle substrate, and this metabolic shift is essential for their responses against infections [184]. Beyond its metabolic role, both endogenous and exogenous lactate promote trained immunity by increasing chromatin accessibility through LDHA-dependent histone lactylation. Supporting this, Ziogas *et al.* provide evidence that lactate production induced by BCG vaccination promotes sustained H3K18 lactylation in monocytes, linking epigenetic memory to enhanced secondary inflammatory responses [183]. Collectively, these findings indicate that lactate serves as a central link between immunometabolic activity and epigenetic regulation in trained immunity.

All the endogenous inducers stimulating trained immunity and their mechanisms are summarized in Table 3.

**Table 3:** Endogenous mediators inducing trained immunity.

**Endogenous Inducers**	**Reported trained cell types**	**Mechanisms**	**Disease model**	**Reference**
Cytokines	See Table 2			
Itaconate, DMI	Monocytes	Mediate by glutathione synthesis	*S. aureus* infection	[169]
Fumarate	Monocytes, macrophages	Inhibit KDM5 histone demethylases, suppress HIF1α degradation, induce mitochondrial fusion	Pharyngeal infection by *E. coli*	[6,7,137,170]
Mevalonate	Monocytes	Activate the cholesterol synthesis pathway, activate IGF1-R and mTOR pathways	HIDS	[30,171]
24(S),25-Epoxycholesterol	Macrophages	Activate LXR signaling, increase glycolysis	Melanoma, Lewis lung carcinoma	[172]
Glucose	Macrophages, monocytes, HSCs	Depend on glycolysis and RUNX1-related epidemic reprogramming	Hyperglycemia-promoted atherosclerosis	[71,74,173]
S100 alarmins	Monocytes	Activate TLR4/MyD88 pathway	Neonatal sepsis	[[175], [176], [177]]
Cathelicidin analogs	Differentiated THP-1 cells	PI3K-mTOR-HIF1α and MAPK p38 pathways	/	[178]
oxLDL	Monocytes, macrophages	Regulated by mTOR-dependent ROS production, activate LXR signaling	Atherosclerosis	[19,179,180]
Aldosterone	Monocyte/macrophages	Upregulate fatty acid synthesis pathway	Cardiovascular diseases	[25,181]
Catecholamine	Monocytes	β-adrenergic receptor-cAMP pathway	PHEO	[25]
Indoxyl sulfate	Monocytes	AhR-AA pathway	CKD	[82]
Lactate	Monocytes, macrophages	LDHA-dependent histone lactylation	*C. albicans* infection	[183,184]

#### Non-canonical trained immunity inducers

(1) Other small-molecule inducers

The trained immunity inducers mentioned above are typically microbial pathogens (*e.g.,* BCG), PAMPs (*e.g.,* β-glucan), or endogenous metabolites. However, small-molecule inducers that are neither pathogen-derived nor disease-related have been rarely reported.

Through high-throughput screening, Knight *et al.* identified glucocorticoids, specifically flunisolide and hydrocortisone which are traditionally considered immunosuppressive agents, as novel inducers of trained immunity [185]. These non-immunogenic glucocorticoids exhibit distinct inflammatory signatures from classical inducers such as β-glucan. Specifically, while they suppress acute inflammation upon initial administration, they subsequently induce a trained phenotype in bone-marrow derived macrophages (BMDMs) in a dose-dependent manner. This indicates that non-immunogenic molecule-induced trained immunity, when employed therapeutically, may not result in adverse inflammatory responses. Further investigations demonstrate that the glucocorticoids-induced trained immunity is dependent on glycolysis and changes in chromatin accessibility. *In vivo* results suggest that flunisolide potentiates TNF-α and IL-6 secretion by increasing the number of eosinophils that responsible for producing these cytokines [185].

In addition to glucocorticoids, Knight *et al.* also identified several other small molecules as trained immunity inducers, including 5-fluoroindole-2-carboxylic acid, myricetin, nerol, hydroquinone and fenoterol hydrobromide*.* Unlike glucocorticoids, these molecules trigger trained immunity independent of glycolytic pathway [185]. Moreover, another study from the same group discovered A1155463, an inhibitor of Bcl-xL, as a novel non-immunogenic trained immunity inducer [186]. Training BMDMs with nanomolar concentrations of A1155463 results in enhanced proinflammatory cytokines production upon LPS challenge, without causing apoptosis. This process is mediated by glycolysis.

Other types of small molecules, including SHIP-1 inhibitors, STING agonists, LXR agonists, RORα antagonists, BIX-01294, oroxylin A, and 17β-estradiol, have also been found to possess capabilities to induce trained immunity [[187], [188], [189], [190], [191], [192], [193]] (Table 4). These findings broaden the spectrum of potential therapeutic agents that modulate trained immunity, although the precise molecular targets and signaling mechanisms by which these compounds induce trained immunity remain to be elucidated.

(2) Other macromolecular inducers

**Table 4:** Non-canonical trained immunity inducers.

**Inducer**	**Reported trained cell types**	**Mechanism**	**Disease model**	**Reference**
Flunisolide, ydrocortisone (Glucocorticoids)	BMDMs	Increase glycolysis	/	[185]
5-fluoroindole-2-carboxylic acid, myricetin, nerol, hydroquinone, fenoterol hydrobromide	BMDMs	/	/	[185]
A1155463 (Bcl-xL inhibitor)	BMDMs	Increase glycolysis	/	[186]
3α-aminocholestane (SHIP-1 inhibitor)	BMDMs, myeloid cells	Activate Akt/mTOR pathway, increase glycolysis	*C. albicans* infection	[187]
DMXAA, c-di-AMP (STING agonists)	Macrophages	Activate Akt/mTOR/HIF1α pathway	*Clostridium perfringens* infection, bladder cancer	[109,188]
T1317, GW3965 (LXR agonists)	Monocytes	Activate mevalonate pathway and IL-1β signaling	/	[189]
SR3335 (RORα antagonists)	Peripheral blood mononuclear cells (PBMCs)	Depend on lactate dehydrogenase A (LDHA) and glycolysis	/	[190]
BIX-01294 (Lysine methyltransferase G9a inhibitor)	Monocytes	/	NMIBC	[191]
Oroxylin A	Macrophages	Increase glycolysis and mTOR phosphorylation, enhance LC3-associated phagocytosis via Dectin-1-syk axis	Sepsis	[192]
17β-Estradiol	Macrophages	Suppress nuclear translocation of RelB to regulate macrophage polarization	Sepsis	[193]
Flagellin	Monocytes, DCs, human sinonasal epithelial cells (HSNECs)	Recognized by TLR5 and NLRC4, activate MyD88 signaling and JNK/MAPK pathways	Chronic rhinosinusitis	[133,194,196]
*S. pneumoniae* endopeptidase O	Macrophages	Upregulate G-CSF expression	Various pathogenic infections	[197]
Co1 peptide (Complement C5a receptor activator)	Macrophages	Activate the mTOR pathway	/	[198]
Leptin	Monocytes	/	Systemic inflammation in subjects with obesity	[199]
Monophosphoryl lipid A (MPLA)	Macrophages	Activate TLR/MyD88 pathway	*S. aureus* infection	[200]
Cholera toxin B subunit	DCs	/	Melanoma	[201]

Flagellin, the principal structural protein of bacterial flagellate filamentum, is a unique PAMP recognized by PRRs such as TLR5 and NLR-family CARD-containing protein 4 (NLRC4), as well as by B and T cell receptors [194,195]. Recognition of flagellin triggers both innate and adaptive immunity, facilitating the rapid elimination of pathogens from the host. Owing to its immunostimulatory properties, flagellin has been extensively investigated as a mucosal adjuvant in vaccine development [194]. Studies have demonstrated that pre-exposure to *P. aeruginosa* flagellin alters the inflammatory responses of bronchial epithelial cells (BECs) upon subsequent exposure to unrelated pathogens or LPS, in a manner dependent on epigenetic regulation [196]. Interestingly, flagellin can induce either trained immunity or tolerance depending on the characteristics and dosage of the secondary stimulus. In another study, initial exposure of monocytes to low concentrations of flagellin increased the response to secondary LPS stimulation, manifested as enhanced secretion of TNF-α and IL-6 [133]. This process is mediated by the p38 and JNK/MAPK signaling pathways, along with epigenetic modifications.

Other macromolecular inducers of trained immunity, such as *S. pneumonia* endopeptidase O, Co1 peptide, leptin, monophosphoryl lipid A (MPLA), and cholera toxin B subunit [[197], [198], [199], [200], [201]], are listed in Table 4. In contrast to nanobiologics, these inducers act directly on cellular signaling or epigenetic pathways without requiring a nanoparticle scaffold or delivery vehicle. Their activity is determined by intrinsic chemical structure rather than carrier-mediated modulation.

### Suppressors of trained immunity

Some modulators of trained immunity can activate innate immune memory, but they elite immune suppression or tolerance upon secondary exposure to identical or different stimuli.

**mTOR inhibitors**

The mTOR/HIF1α axis-dependent glycolysis serves as the metabolic basis for the establishment of trained immunity [7]. Rapamycin, a potent mTOR inhibitor with significant immunosuppressive effects, is clinically used to prevent organ transplant rejection and treat autoimmune diseases. Cheng *et al.* demonstrated that rapamycin effectively inhibits β-glucan induced trained immunity [7]. Similarly, metformin, which activates AMPK and inhibits mTOR, has been shown to suppress trained immunity *in vitro* and reduce the protective effects against *C. albicans* infection *in vivo* [7].

At the mechanistic level, mTOR serves as a key sensor of the cellular metabolic state, orchestrating glucose metabolism and coordinating immune cell activation. Its inhibition suppresses HIF1α expression, thereby downregulating metabolic pathways such as aerobic glycolysis and the TCA cycle. These pathways generate metabolic intermediates such as acetyl-CoA and fumarate, which are critical for regulating histone modifications (*e.g.*, acetylation and methylation) through modulation of enzymes such as histone acetyltransferases (HATs) and histone demethylases (*e.g.*, KDM5). As a consequence, reduced mTOR signaling lowers histone acetylation (*e.g.*, H3K27ac) and limits histone demethylation (*e.g.*, H3K4me3), leading to the repression of pro-inflammatory gene transcription and cellular activation programs associated with trained immunity [6].

Moreover, rapamycin and related mTOR inhibitors have been shown to decrease the deposition of activating histone marks such as H3K4me3 and H3K27ac at promoters of inflammatory genes, thereby directly attenuating the enhanced transcriptional responsiveness of monocytes/macrophages upon secondary stimulation [6,30]. Together, these findings support that mTOR inhibitors suppress trained immunity not only by altering metabolic pathways but also through shaping specific epigenetic landscapes, particularly histone methylation and acetylation, which control long-term transcriptional memory in innate immune cells.

Braza *et al.* developed a myeloid cell-specific nanoimmunotherapy based on mTOR inhibitor-high-density lipoprotein (mTORi-HDL), in which rapamycin is encapsulated in HDL nanoparticles [80]. Through H3K4me3 epigenetic modifications, this formulation effectively suppresses trained immunity, as evidenced by the inhibition of aerobic glycolysis and reduced release of inflammatory cytokines in macrophages. These trained macrophages not only block alloreactive CD8^+^ T cell-mediated responses but also facilitate the proliferation of tolerogenic CD4^+^ regulatory T cells (Tregs). Notably, the combination of mTORi-HDL treatment with TRAF6i-HDL nanoimmunotherapy, a co-stimulation inhibitor targeting CD40, results in long-term allograft survival [80]. This innovative translational therapy demonstrates potential for improving organ transplant success while minimizing the need for sustained immunosuppression.

**Anti-inflammatory cytokines**

Anti-inflammatory cytokines such as IL-10 and IL-35 exert crucial functions in immune tolerance and regulation of immune functions, with evidence showing that they inhibit acute activation of human aortic endothelial cells (HAECs) [163]. Among them, IL-35 demonstrates superior efficacy compared to IL-10 in inhibiting lipid lysophosphatidylcholine (LPC)-triggered innate immune gene expression in HAECs. LPC activates innate immunity through metabolic regulation of chromatin reprogramming and mitochondrial ROS regulation of transcriptional factors. Mechanistically, neither IL-10 nor IL-35 suppresses the reprogramming of crucial immunometabolic pathways induced by LPC; however, they partially suppress LPC-induced innate immunity via inhibiting mitochondrial ROS production [163]. Additionally, Röring *et al.* provide evidence that IL-10 suppresses trained immune responses in primary monocytic cells mainly by inhibiting STAT3 signaling pathway and ROS production [202]. Furthermore, IL-10 downregulates glycolytic and oxidative metabolism but does not alter the metabolic signatures of β-glucan-induced trained immunity.

IL-37 and IL-38, two anti-inflammatory cytokines within the IL-1 family, have been demonstrated to abrogate the effect of trained immunity induced by β-glucan via blocking mTOR signaling *in vivo*. This is manifested by decreased generation of inflammatory mediators, reversal glycolysis reprogramming, and mitigation of epigenetic changes in BMDMs induced by β-glucan [165,166]. IL-37 activates AMPK and subsequently suppresses mTOR. Furthermore, it directly inhibits IL-1β processing by blocking ASC oligomerization [203]. IL-38 has been reported to prevent β-glucan–induced trained immunity and reverse the increase in H3K4me3-positive promoters observed in β-glucan-trained mice. IL-38 disrupts AKT/mTOR/S6K signaling in BMDMs and also downregulates HIF1α and IL-1β in synovial tissue cells. Moreover, the *IL1F10*-related SNP rs58965312 correlates with elevated plasma IL-38 levels and weakened β-glucan-induced trained immunity responses *ex vivo* [166]. These findings suggest potential applications of IL-37 and IL-38 in treating cardiovascular disorders, chronic inflammatory conditions, and malignancies, although further research is needed to elucidate their precise roles in trained immunity.

**MyD88 inhibitors**

MyD88-dependent signaling is essential for TLR-induced trained immunity [200]. Its inhibition has been shown to ameliorate transplantation rejection. Treatment with TJ-M2010-5, a small-molecule MyD88 inhibitor, has been demonstrated to prolong xenograft survival in mice while suppressing systemic inflammation in xenograft recipients (SIXR) and mitigating acute vascular rejection (AVR) [204]. Mechanistically, TJ-M2010-5 impedes CCL-elicited nuclear translocation and phosphorylation of NF-κB p65 in BMDMs and bone marrow-derived dendritic cells (BMDCs), while also prevents epigenetic modifications (*e.g.*, H3K4me3) at pro-inflammatory cytokine loci such as IL-6 and TNF-α. Moreover, MyD88 inhibition leads to downregulation of co-stimulatory markers CD80 and CD86, thereby reducing downstream innate immune responses [204]. Combining TJ-M2010-5 with an anti-CD154 antibody further augments the inhibitory effect on AVR and SIXR, thus prolonging graft survival.

**Other types of trained immunity suppressors**

The therapeutic application of chloroquine and hydroxychloroquine for managing SARS-CoV-2 infections remains controversial due to uncertainties surrounding their efficacy and mechanisms of action. Rother *et al.* demonstrated that both drugs diminish the generation of IL-6 and TNF-α in peripheral blood mononuclear cells (PBMCs) trained with heat-killed *C. albicans* (HKCA) and subsequently restimulated with LPS or Pam3CSK4 *in vitro* [205]. Hydroxychloroquine also inhibits the expression of genes stimulated by interferons and prevents histone modifications such as H3K27 acetylation and H3K4 trimethylation. Due to its alkaline side chain, hydroxychloroquine accumulates in lysosomes, potentially impairing lysosomal acidification and modulating mTOR signaling, which consequently suppresses trained immunity [205]. Furthermore, hydroxychloroquine alters cellular lipid metabolism by preventing the upregulation of phosphatidylinositol (PI) and phosphatidylserine (PS) lipids, further contributing to its immune-suppressive profile [205]. These findings indicate that hydroxychloroquine may have detrimental effects on the antiviral immune responses required for host defense against SARS-CoV-2 infection.

LXRs serve as essential regulators of cholesterol homeostasis and fatty acid metabolism. While LXR activation enhances BCG-induced trained immunity [189], LXR antagonism inhibits it. Findeisen *et al.* demonstrated that the LXR antagonist GSK2033 suppresses oxidized LDL-triggered trained immunity in monocytes by inhibiting aerobic glycolysis, cholesterol efflux, and fatty acid synthesis pathways [180]. Furthermore, it reduces histone alterations, including H3K27ac and H3Kme3 at the *IL-6* and *TNFα* promotors.

Other types of molecules, including itaconate, I-BET151, dihydroartemisinin, vitamin A, FhHDM-1, and short-chain fatty acids, have also been found to suppress trained immunity [168,174,[206], [207], [208], [209]] (Table 5). However, the specific molecular targets and signaling pathways by which these molecules suppress trained immune responses remain insufficiently explored. Furthermore, certain molecules such as resveratrol exhibit controversial effects on trained immunity, highlighting the need for further investigation into their immunological roles [210].

**Table 5:** Modulators suppressing trained immunity.

**Inhibitor**	**Reported trained cell types**	**Mechanism**	**Disease model**	**Reference**
Rapamycin, Metformin, mTORi-HDL (mTOR inhibitors)	Monocytes, macrophage	Inhibit the mTOR pathway	*C. albicans* infection, organ transplantation	[7,80]
IL-10, IL-35, IL-37, IL-38 (anti-inflammatory cytokines)	See Table 2			
TJ-M2010-5 (MyD88 inhibitor)	BMDCs, BMDMs	Block MyD88/NF-κB signaling pathway, reduce the expression of CD80/CD86	Xenotransplantation model	[204]
GSK2033 (LXR antagonist)	Monocytes, macrophages	Inhibit LXR signaling pathway	/	[180]
Itaconate, DMI (under acute inflammatory condition)	Monocytes	Block SDH activity and inhibit TCA cycle	Endotoxemia	[168]
Hydroxychloroquine	Monocytes	Inhibit trained immunity-induced alterations in lipidome and histone modifications	SARS-CoV-2 infection, heat-killed *C. albicans* challenge	[205]
I-BET151 (Bromodomain inhibitor)	Monocytes	/	*C. albicans* infection	[206]
Dihydroartemisinin	BMDMs	Suppress the activation of AKT/mTOR/HIF1α pathway	Heat-killed *C. albicans* challenge	[207]
Vitamin A	Monocytes, macrophages	Increase H3K9me3 and activate the histone methyltransferase SUV39H2	/	[208]
FhHDM-1	Macrophages	Inhibit histone methylation and glycolysis	Non-obese diabetes mellitus	[174]
Short-chain fatty acids	Monocytes, DCs	/	/	[209]

**Fig. 4:**
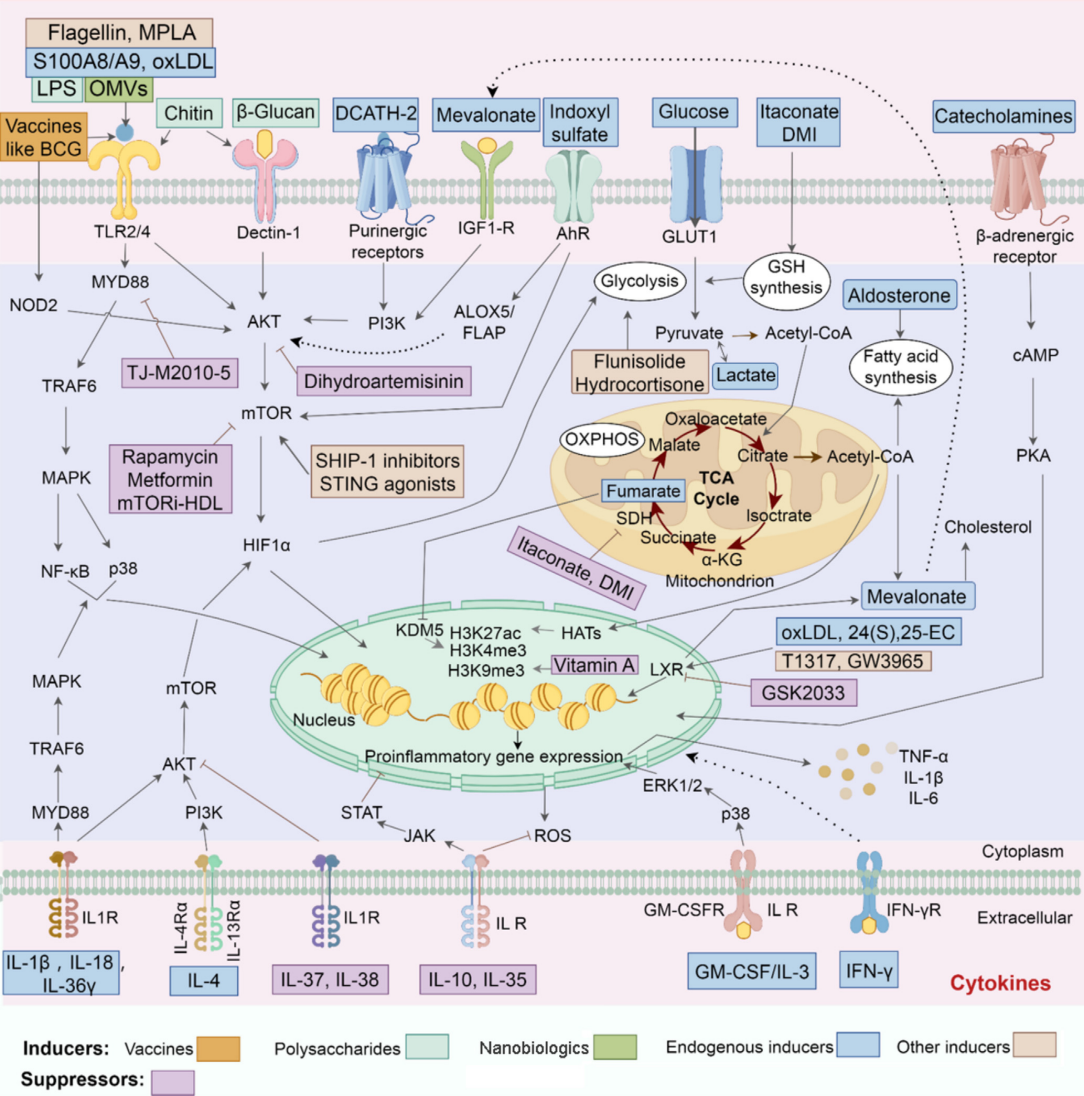
**Trained immunity modulators and their associated signaling pathways**. Initial exposure to inducers (*e.g.*, vaccines, polysaccharides, nanobiologics) initiates a succession of intracellular cascades that upregulate various metabolic pathways. The endogenous metabolites, such as fumarate, glucose and certain cytokines, also serve as pivotal regulators in shaping trained immune responses. Suppressors that downregulate those metabolic pathways or block chromatin accessibility inhibit trained immunity. The figure is created with Figdraw.com.

Taken together, we present a comprehensive overview of the classifications and mechanisms of trained immunity inducers and suppressors identified over the past decade, including vaccines, polysaccharides, nanobiologics, endogenous mediators and other non-canonical modulators, with emphasis on their therapeutic potential in immune-related diseases. The trained immunity modulators and their associated signaling pathways are summarized in Fig. 4.

## Prospects and Conclusions

Trained immunity is a novel concept that has recently emerged, suggesting that innate immune cells possess memory properties. These cells undergo functional remodeling in response to primary exogenous or endogenous stimuli, leading to durable functional changes and enhanced inflammatory responses upon subsequent stimulation. Central mechanisms of trained immunity include metabolic alterations and epigenetic reprogramming, with ongoing efforts to elucidate the pathways and mechanisms involved. However, this fascinating new field of immunology still presents numerous questions warranting deeper explored. For example, the molecular mechanisms mediating trained immunity require more detailed characterization, including comprehensive investigations into both immune cell and non-immune cell populations capable of inducing trained immunity. Additionally, there is a need for refined insights into the metabolic, epigenetic and immunological processes that involved in trained immunity. Another critical research priority involves understanding the molecular mechanisms regulating interactions between innate and adaptive immunological mechanisms. The combination of trained immunity inducers with adaptive immunotherapy approaches (such as vaccines and immune checkpoint blockade therapies) may yield synergistic or unpredictable effects. These interactions could either enhance the efficacy of treatment or, conversely, increase the risk of adverse effects, thereby necessitating detailed mechanistic and translational studies. Comprehensive studies in these areas will not only deepen our understanding of trained immunity but also provide crucial insights for designing targeted therapeutic strategies to regulate trained immune responses.

Other fascinating areas deserving further research include whether trained immunity can be inherited epigenetically across generations, and the impact of host microbiota on trained immune responses. Epigenetic reprogramming caused by trained immunity may have unpredictable long-term consequences. These alterations could potentially be inherited by future generations (epigenetic inheritance). While such transmission may confer benefits in certain contexts, it also raises concerns about unexpected genetic or epigenetic effects that may affect offspring. Moreover, the host microbiome serves as a key regulator in trained immunity modulation. However, the dynamic interactions between the microbiome and immune system complicate the use of trained immunity modulators. Variability in the microbiome between individuals could generate inconsistent responses, thereby limiting the general application of these immunomodulators.

Trained immunity has profound implications for disease prevention and therapeutic interventions that involve the immune system, including infections, cancers, and chronic inflammatory conditions. Traditional vaccines primarily work by stimulating the adaptive immune system, but trained immunity can be used to enhance the first line of defense in innate immune system. Trained immunity-promoting vaccines offer broader and faster protection against diverse pathogenic microorganisms, including bacteria, fungi and viruses, which could be particularly beneficial in preventing cross-infections. For instance, the BCG vaccine has been shown to confer non-specific protection against respiratory viral infections and certain malignancies through the induction of trained immunity [47,48,211].

Rather than relying solely on traditional immunization, developing therapies that specifically enhance trained immunity may be particularly valuable for vulnerable populations, such as the elderly and immunocompromised individuals, who tend to have weaker adaptive immune responses. Trained immunity-boosting immunotherapies can help combat persistent or recurrent infections, especially those caused by multidrug-resistant bacteria and fungi, and may also reduce the incidence of certain types of cancers. These strategies are largely beneficial in reducing the healthcare burden.

This review provides a comprehensive and up-to-date summary of trained immunity modulators (Fig. 3, Fig. 4). Although many types of them show promising potential for use in various therapeutic settings, some limitations still remain, including:

(1) Druggability and safety. Most of the well-known trained immunity inducers reported to date are PAMPs, which are conserved molecules present in different species, such as polysaccharides derived from pathogenic microorganisms and plants. They are generally recognized by PRRs on the surface of a variety of specific immune and non-immune cells. However, the nonspecific nature of this recognition might lead to unwanted immune responses or a broad-spectrum activation of the immune system, potentially causing adverse effects or aggravating autoimmune diseases. Furthermore, although polysaccharides like β-glucan exhibit certain potential in the treatment of infections and cancers, several challenges remain, including issues related to low bioavailability, standardized production and quality control, and the safety of long-term use [121]. Future advancements in drug delivery technologies and additional clinical studies are needed to reduce these risks, making β-glucan a promising therapeutic agent in treating immune-related diseases.
(2) Duration of trained immunity. Even though trained immunity is long-lasting, it is not permanent. A comprehensive elucidation of the duration of trained immunity triggered by various stimuli, including vaccines, polysaccharides and nanobiologics, is essential. This impermanence represents an opportunity as well as a challenge. Pharmacologically, temporarily triggering trained immunity could overcome immune paralysis caused by infections, cancers or sepsis [16,128,156,211]. Meanwhile, once the disease is under control, it is crucial to mitigate the risk of long-term chronic inflammation by suppressing exogenously triggered trained immunity. On the contrary, for conditions such as organ transplantation, where suppressing trained immunity is beneficial, treatment should be discontinued once the therapeutic effect is achieved.
(3) Risk of overstimulation and dysregulation of immune responses. Prolonged or inappropriate stimulation of trained immunity by inducers may result in excessive inflammatory responses or immune activation. This overstimulation might potentially increase the risk of autoimmune diseases or chronic inflammation. Therefore, when using trained immunity inducers, a balance should be struck between beneficial trained immune responses and preventing harmful immune responses. Due to the fact that trained immunity is contingent upon alterations in innate immune cell functional phenotypes, it may inadvertently lead to immune response dysregulation, such as inability to respond adequately to new pathogens or inappropriate immune responses to self-tissues. Some non-immunogenic compounds, such as glucocorticoids, have created novel avenues for the study and application of trained immunity, which can induce trained immunity without triggering inflammatory responses, or have a window period of active suppression of inflammation under acute inflammatory conditions [185]. However, the efficacy and mechanisms of these compounds in the treatment of related diseases through trained immunity remain to be fully elucidated. Natural products represent a major source of trained immunity modulators. In addition to the agents summarized in this review, several other molecules—such as certain natural host defense peptides and tumor-associated factors—also play roles in regulating innate immune responses [212,213]. However, whether these molecules can drive the establishment of memory-like features in innate immune cells requires further investigation.

In conclusion, trained immunity is an attractive and constantly evolving concept in immunology, with profound influence on both health and disease. The recognition that innate immune cells, especially monocytic cells, macrophages and neutrophils, can undergo long-term functional reprogramming to generate memory under environmental stimulation, providing new avenues of therapeutic strategies for diseases like infections, cancers, and organ transplantation. Although the potential application of trained immunity is enormous, there are still considerable challenges in fully clarifying the potential mechanisms, optimizing the immunomodulators for clinical use, and ensuring their safety and efficacy in humans. The complicated interactions between trained immunity and adaptive immunity, and the influence of genetic predisposition and environmental determinants, should be further investigated to optimize the therapeutic benefit. Future research is essential for improving our knowledge of trained immunity, identifying more specific immunomodulators, and exploring their applications in different clinical settings. With continuous progress, trained immunity may become an indispensable part of personalized medicine, opening the way for new therapies for infectious diseases, cancers, and even autoimmune diseases.

## Declaration of competing interest

The authors declare that they have no known competing financial interests or personal relationships that could have appeared to influence the work reported in this paper.
